# Diabetes insipidus and panhypopituitarism as a first presentation of silent adenocarcinoma of lung: a case report and literature review

**DOI:** 10.1186/s12902-019-0445-5

**Published:** 2019-10-29

**Authors:** Sirinart Sirinvaravong, Peeradon Vibhatavata, Paweena Chunharojrith, Pornsuk Cheunsuchon, Sutin Sriussadaporn

**Affiliations:** 1 Division of Endocrinology and Metabolism, Department of Medicine, Faculty of Medicine Siriraj Hospital, Mahidol University, Bangkok, Thailand; 2 Department of Pathology, Faculty of Medicine Siriraj Hospital, Mahidol University, Bangkok, Thailand

**Keywords:** Pituitary metastasis, Diabetes insipidus, Panhypopituitarism, Adenocarcinoma of lung

## Abstract

**Background:**

Pituitary metastasis is a rare condition with a poor prognosis. Very few patients with pituitary metastasis are symptomatic. It is often associated with presence of co-existing metastases to other organs. Isolated pituitary metastasis as the first presentation of primary malignancy is uncommon.

**Case presentation:**

A 72-year-old woman presented with a 2-month history of polyuria, increasing thirst and unexplained weight loss. Esophagogastroduodenoscopy (EGD) was scheduled as part of the investigation. She was kept *nil per os* for 10 h prior to EGD, after which she developed alteration of consciousness. Further investigation revealed hypernatremia with sodium level of 161 mmol/L and low urine osmolality of 62 mOsm/kg. Her urine output was 300 mL per hour. Diabetes insipidus (DI) was diagnosed based on evidence of polyuria, hypernatremia, and low urine osmolality. Her urine output decreased and urine osmolality increased to 570 mOsm/kg in response to subcutaneous desmopressin acetate, confirming central DI. Pituitary magnetic resonance imaging showed a heterogeneous gadolinium enhancing lesion at the sellar and suprasellar regions, measuring 2.4 × 2.6 × 3.9 cm compressing both the hypothalamus bilaterally and the inferior aspect of optic chiasm as well as displacing the residual pituitary gland anteriorly. The posterior pituitary bright spot was absent. These MRI findings suggested pituitary macroadenoma. There were also multiple small gadolinium-enhancing lesions up to 0.7 cm in size with adjacent vasogenic brain edema at the subcortical and subpial regions of the left frontal and parietal areas, raising the concern of brain metastases. Pituitary hormonal evaluation was consistent with panhypopituitarism. Histopathological and immunohistochemical studies of the pituitary tissue revealed an adenocarcinoma, originating from the lung. Computed tomography of the chest and abdomen was subsequently performed, showing a 2.2-cm soft tissue mass at the proximal part of right bronchus. There was no evidence of distant metastases elsewhere. The final diagnosis was adenocarcinoma of the lung with pituitary metastasis manifesting as panhypopituitarism and central DI. Palliative care along with hormonal replacement therapy was offered to the patient. She died 4 months after diagnosis.

**Conclusion:**

Diagnosis of pituitary metastasis is challenging, especially in patients with previously undiagnosed primary cancer. It should be considered in the elderly patients presenting with new-onset central DI with or without anterior pituitary dysfunction.

## Background

Metastasis to pituitary gland is a rare situation in clinical practice with a poor prognosis. The prevalence of metastatic pituitary tumors was approximately 1% among all pituitary tumor resections [1] and 1 to 3.6% among post-mortem studies [2]. Most of them were asymptomatic and were typically detected incidentally on imaging or at autopsy [3]. One series found that only 7% of patients with pituitary metastases were symptomatic [4]. Symptomatic pituitary metastasis as the first presentation of primary tumor is uncommon.

Pituitary metastasis is often associated with the presence of multiple additional metastatic sites, especially in bones [5, 6]. Isolated pituitary metastasis is rare [7–15], and its clinical presentations, pituitary hormonal profiles, and radiological imaging features mimic those of the more common primary pituitary tumors [16]. These characteristics may lead to misdiagnosis and delayed treatment, especially in individuals without a known pre-existing malignancy. Here, we report a rare case of isolated pituitary metastasis from adenocarcinoma of the lung first presenting as central diabetes insipidus and panhypopituitarism without known evidence of the primary malignancy. A literature review was performed. A search of the literature was performed on the PubMed and Ovid Medline databases. The initial search string used was “Pituitary AND Metastas.*” References cited in the articles identified by our original search were also assessed for relevance. Most of the papers identified were case studies and case series; those in languages other than English were excluded.

## Case presentation

**Fig. 1:**
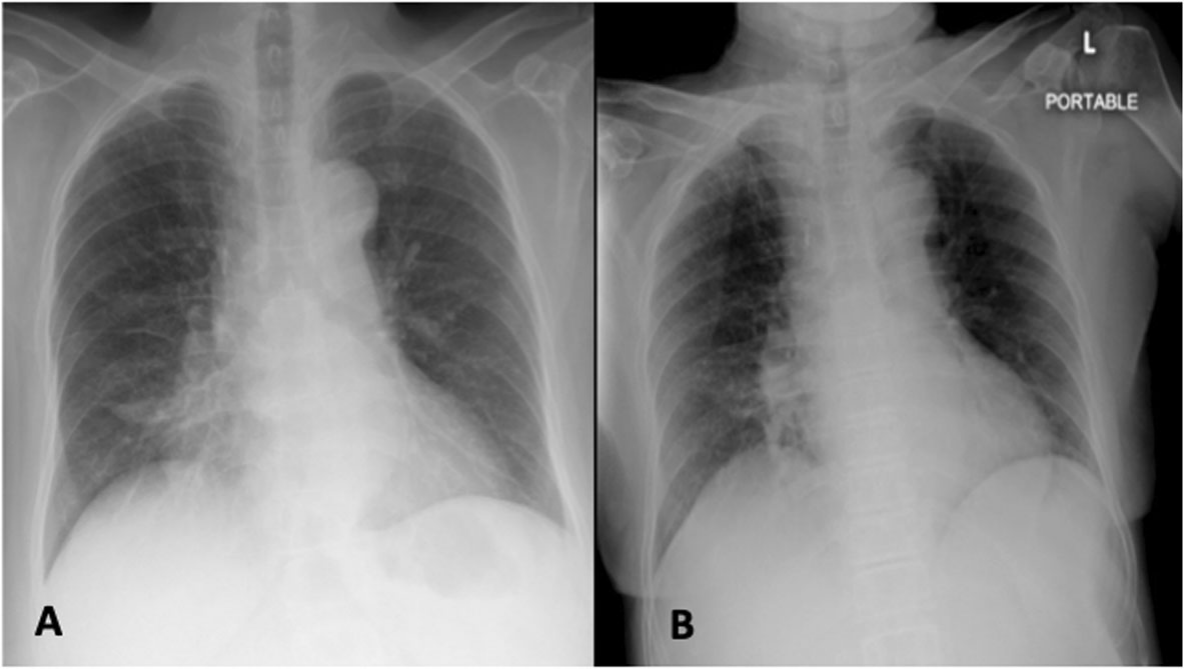
Chest radiograph 3 months prior to admission showing right middle lobe pneumonia (**a**) and on admission showing minimal infiltration at medial aspect of right lower lung (**b**)

A 72-year-old woman with a 5-pack-year smoking history presented with drowsiness after esophagogastroduodenoscopy (EGD). Her past medical history was remarkable for community-acquired pneumonia at right middle lung (Fig. 1a) 3 months prior, from which she made a full recovery following antibiotic therapy. She had a 2-month history of polyuria, polydipsia, nocturia, lightheadedness upon standing up quickly, fatigue, loss of appetite, and 15 kg of weight loss from a baseline weight of 60 kg. She had no fever, cough, dyspnea, hemoptysis, chest pain, or night sweats. She presented to the outpatient unit, and the investigation at that time showed a fasting plasma glucose level of 100 mg/dL (5.5 mmol/L) and an HbA1c of 4.8%. Thyroid function tests revealed a normal serum triiodothyronine (T3) level of 2 nmol/L (reference range 1.23–3.07 nmol/L), a low free thyroxine (FT4) level of 3.86 pmol/L (reference range 11.97–21.88 pmol/L), and a suppressed thyroid stimulating hormone (TSH) level of 0.06 mIU/L (reference range 0.27–4.0 mIU/L). A chest x-ray showed minimal infiltration at medial aspect of right lower lung, which had decreased compared with a previous chest x-ray (Fig. 1b).

The patient underwent EGD 2 months after the outpatient visit. She was kept *nil* *per os* for 10 h prior to the procedure. The EGD findings were mild non-erosive antral gastritis. After EGD, she developed nausea, vomiting, and drowsiness. Physical examination revealed a body temperature of 37 °C, a blood pressure of 100/57 mmHg, a pulse rate of 90/min, and a respiratory rate of 16/min. She was 44.5 kg in body weight, was 148 cm in height, and had a body mass index of 20.3 kg/m^2^. She had flat neck veins, a normal thyroid gland without nodules, normal breath sounds, no abnormal palpable masses, no hepatosplenomegaly, no breast masses, and no superficial lymphadenopathy. Neurological examination was remarkable for bitemporal hemianopia evaluated by confrontation test.

She was immediately admitted to the hospital because of the altered mental status. At the first hour after admission, she had polyuria with a urine output of 300 mL/hour (6.7 mL/kg/hour). Laboratory tests showed a serum sodium level of 160 mmol/L; a potassium level of 3.9 mmol/L; a chloride level of 125 mmol/L; a bicarbonate level of 24 mmol/L; a creatinine level of 1.4 mg/dL. Serum osmolality was 325 mOsm/kg. Her urine specific gravity was 1.002 without proteinuria or glucosuria. Urine osmolality was 62 mOsm/kg. Diabetes insipidus was diagnosed based on evidence of polyuria along with hypernatremia and low urine osmolality. Desmopressin acetate (DDAVP) 1 microgram was given by subcutaneous injection. One hour later, her urine output decreased to 70 mL/hour, and urine osmolarity increased to 570 mOsm/kg. Based on decreasing urine output and a more than 50% increase in urine osmolality in response to DDAVP, a diagnosis of central diabetes insipidus was made.

**Fig. 2:**
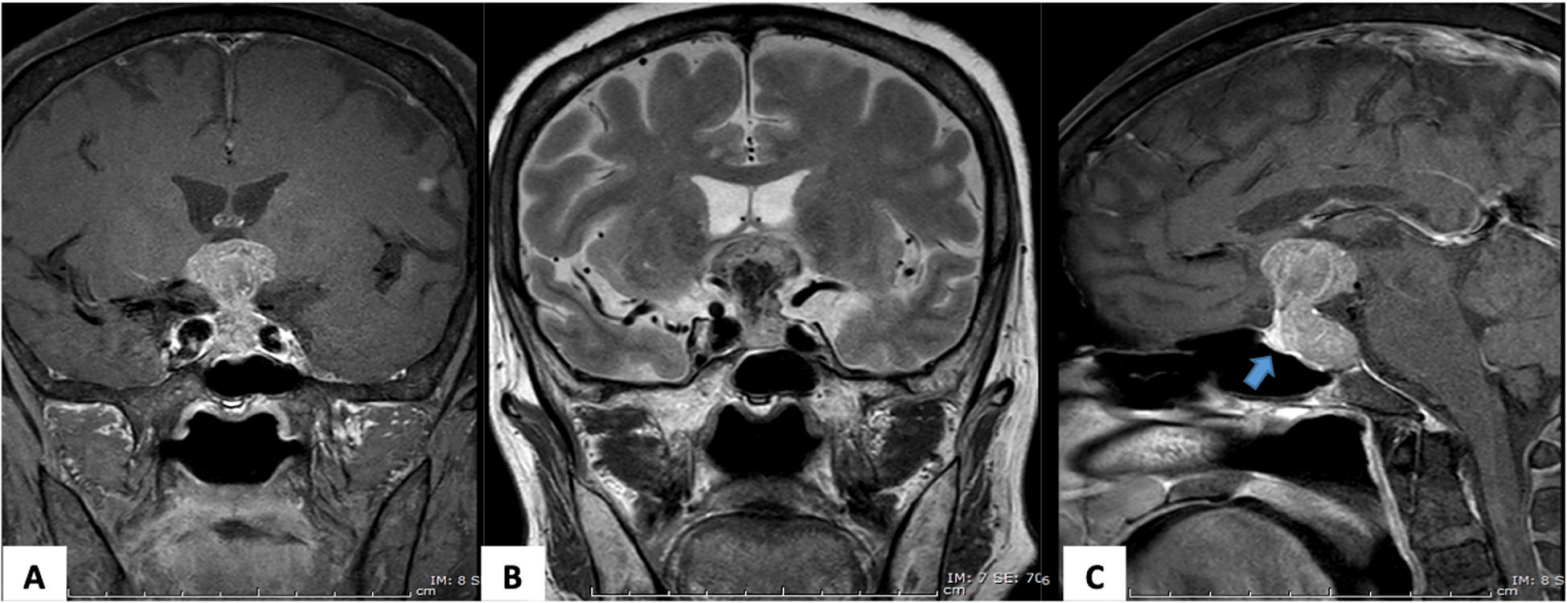
Pituitary MRI with gadolinium contrast. Coronal T1W (**a**), coronal T2W (**b**), sagittal T1W (**c**) images showing a heterogeneous enhancing lesion at sellar and suprasellar regions with a high signal intensity on T1W imaging and a low signal intensity on T2W imaging with central necrosis, abutting the medial aspect of bilateral cavernous sinus. The lesion was compressing the inferior aspect of optic chiasm and anteriorly displacing the residual pituitary gland (arrow)

**Fig. 3:**
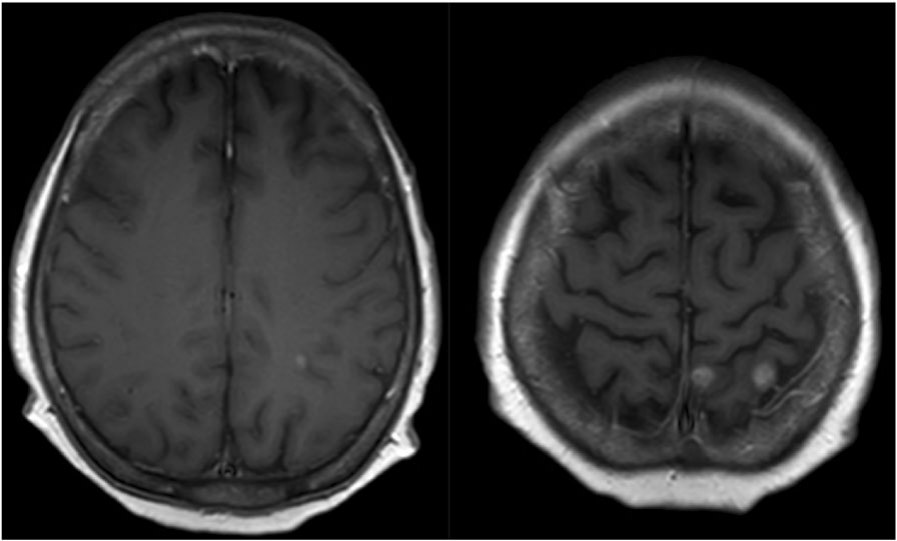
Multiple small gadolinium enhancing lesions with adjacent vasogenic edema at the subcortical region of the left frontal and parietal areas shown on T1W axial images of the pituitary MRI

Given the diagnosis of central diabetes insipidus, further investigations including magnetic resonance imaging (MRI) of the pituitary gland and evaluation of the anterior pituitary hormones were performed. Pituitary MRI demonstrated a heterogeneous high signal intensity (SI) lesion in T1-weighted (T1W) imaging, which also appeared as a low SI lesion in T2-weighted (T2W) imaging with heterogeneous gadolinium enhancement at the sellar and suprasellar region, measuring 2.4 × 2.6 × 3.9 cm. The lesion was compressing the hypothalamus bilaterally and the inferior aspect of optic chiasm with increased SI in T2W imaging at the right optic nerve and bilateral optic tracts. It was also anteriorly displacing the residual pituitary gland. The pituitary stalk could not be identified, and the bright spot of posterior lobe was absent. These MRI findings suggested pituitary macroadenoma with hemorrhage (Fig. 2). The bony skull showed a normal appearance without lytic lesion. There were also multiple small gadolinium-enhancing lesions up to 0.7 cm in size with adjacent vasogenic brain edema at subcortical and subpial regions of the left frontal and parietal areas; therefore, multiple stages of cysticercosis and brain metastases were included in the differential diagnosis (Fig. 3).

Pituitary hormonal profiles (Table 1) showed inappropriately low gonadotropins levels, a low plasma adrenocorticotropic hormone (ACTH) level, and a low morning serum cortisol level that did not respond to cosyntropin stimulation, indicating secondary adrenal insufficiency. Serum prolactin level was mildly elevated, but no further increase was seen in a diluted sample. Hyperprolactinemia was suspected to be caused by stalk interruption. Thyroid function tests performed 2 months prior to admission showed a low free T4 level of 3.86 pmol/L and a low TSH level of 0.06 mIU/L, suggesting central hypothyroidism. On admission, serum free T4 level had decreased to 2.32 pmol/L with an unexpectedly elevated TSH level of 19.07 mIU/L. A low IGF-1 level in combination with deficiency of more than two pituitary hormones was highly indicative of growth hormone deficiency.

**Table 1:** Initial hormonal evaluation

Serum hormone (SI unit)	Result	Reference range
IGF-1 (nmol/L)	2.69	3.01–27.32
Prolactin (μg/L)	115	4.79–23.3
Prolactin after 1:100 dilution (μg/L)	81	4.79–23.3
FSH (IU/L)	1.39	25.8–134.8
LH (IU/L)	<0.1	7.7–58.5
FT4 (pmol/L)	2.32	11.97–21.88
TSH (mIU/L)	19.07	0.27–4.2
ACTH (ng/L)	26.3	10.0–60.0
Morning cortisol (nmol/L)	138	
Cortisol after 250 μg ACTH stimulation test (nmol/L)		
at 30 min	237.3	
at 60 min	306.2	

*IGF-1* Insulin-like growth factor 1, *FSH* Follicular stimulating hormone, *LH* Luteinizing hormone, *FT4* Free thyroxine, *TSH* Thyroid-stimulating hormone, *ACTH* Adrenocorticotropic hormone

**Fig. 4:**
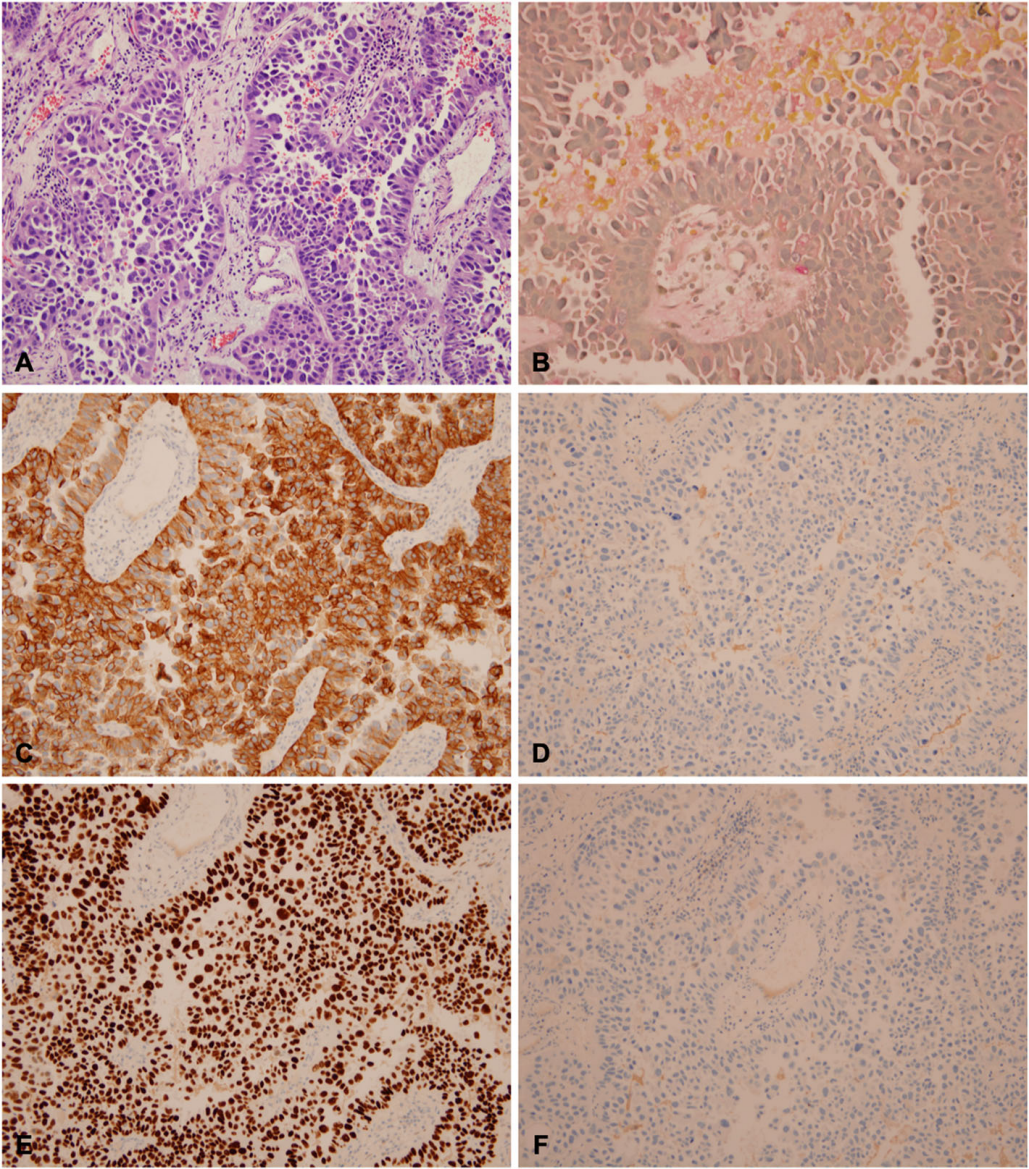
Tumor resected from sellar mass composing of malignant epithelial cells with glandular formation (**a** hematoxylin-eosin, × 100) and intracellular mucin (**b** × 200). Immunohistochemical study showed that tumor cells were immunoreactive for antibodies of cytokeratin 7 (**c** × 200) and TTF-1 (**e** × 200) but not cytokeratin 20 (**d** × 200) and CDX-2 (**f**, × 200)

The patient underwent craniotomy for tumor removal. Intraoperative findings showed one grey-purplish firm mass at the sellar and retrochiasmatic regions with some necrosis and hemorrhage. The tumor was partially removed. A histopathological study revealed adenocarcinoma with the immunohistochemical staining positive for cytokeratin-7 and thyroid transcription factor-1 (TTF-1) but negative for cytokeratin-20, CDX-2 and GFAP (Fig. 4). These findings were consistent with metastatic adenocarcinoma originating from the lung.

**Fig. 5:**
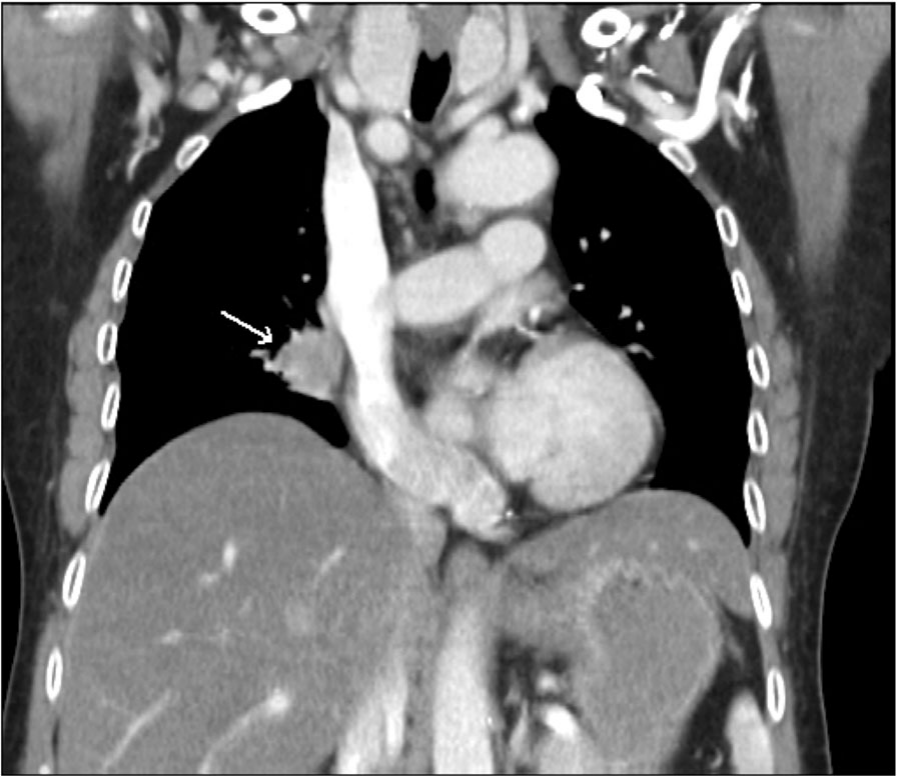
CT chest with contrast revealing an enhancing soft tissue mass occupying the proximal part of bronchus supplying the medial segment of right middle lung (arrow)

To identify the primary adenocarcinoma of the lung, a CT scan of the chest was performed which revealed a 2.2 × 2.2 cm enhancing soft tissue mass at the proximal part of the right bronchus that supplies the medial segment of the right middle lung. This was causing atelectasis of the distal part of the medial segment of the right middle lung and diffuse centrilobular emphysema at both upper lobes without pleural effusion. The liver, spleen, kidneys, and adrenal glands appeared unremarkable. No osteolytic lesion of the ribs, spine, and other bony structures were seen (Fig. 5). The final diagnosis was advanced adenocarcinoma of the lung with pituitary metastasis associated with panhypopituitarism and central diabetes insipidus.

Palliative care for the advanced-stage lung cancer was offered to the patient. She received hormone replacement including desmopressin acetate 10 mcg intranasal solution daily, oral prednisolone 5 mg daily, and levothyroxine 75 mcg daily for central diabetes insipidus, secondary adrenal insufficiency, and central hypothyroidism, respectively. Two months later, she developed bilateral ophthalmoplegia and died 4 months after the diagnosis.

## Discussion and conclusions

Pituitary metastases are usually detected in patients with advanced cancer and associated with widespread metastases, typically affecting elderly patients, especially those aged 60–70 years [17]. They often remain silent because co-existing metastases to the other organs cause death first [18]. According to one autopsy series, the prevalence of pituitary metastases was 0.14 to 28.1% among all brain metastases [19]. Latent pituitary metastases were found in 5% of patients with known primary malignancies, of which around two-thirds had macroscopically normal pituitary glands [4]. Malignant neoplasms of all tissue types from nearly all organs have been reported to metastasize to the pituitary. However, breast and lung are the most common sites of the primary tumor, accounting for 39.7 and 23.7% of all pituitary metastases, respectively [1]. A Swedish population-based study of metastatic sites of lung cancer reported that among primary lung cancers with pituitary metastasis, small cell lung cancer is the most common cell type, whereas adenocarcinoma and squamous cell carcinoma rarely metastasize to the pituitary gland but rather to the bones and respiratory system. Only a few cases of adenocarcinoma of the lung metastasized to the pituitary gland without other distant metastases [20]. Unlike typical cases, our patient had adenocarcinoma of the lung and the first manifestation was central diabetes insipidus and hypopituitarism secondary to pituitary metastasis without evidence of primary lung cancer or widespread metastasis.

The four pathways of metastases to the pituitary gland [21–23] include direct hematogenous spreading to the pituitary parenchyma or diaphragma sellae, spreading from a hypothalamo-hypophyseal or infundibulum metastasis through the portal vessels, extension from juxtasellar and skull base metastasis, and meningeal spreading through the suprasellar cistern.

Regarding the location of metastatic tumors in the pituitary, 84.6% were found at the posterior pituitary either alone or in combination with the anterior pituitary whereas only 15.4% were found at the anterior pituitary alone [1]. The posterior pituitary is a preferential site for metastases as it receives direct arterial blood supply from hypophyseal arteries whereas the blood supply to the anterior pituitary is mainly from the hypophyseal portal system, which is not arterial. Thus, the anterior pituitary is less predisposed to pituitary metastasis than the posterior portion [1, 23]. Also, the posterior lobe has a larger contact area with dura mater [4, 5]. Metastases to the anterior pituitary mostly originate from direct spreading of metastatic foci from the posterior pituitary [1] and associate with a larger area of the extending posterior pituitary lesion. In addition, the tumor in posterior lobe or stalk may potentially compromise a blood supply of the anterior lobe, resulting in ischemic infarct [4]. Thus, involvement of the anterior pituitary in our patient could be explained by the effect of large metastatic volume in the posterior pituitary as well as a damage to the vascular supply.

The majority of pituitary metastases are clinically silent. Symptomatic pituitary metastases were found in only 7% of cases [4], and it was rarely the first manifestation of the cancer [24]. Based on a literature review between 1994 and 2018, 26 cases of symptomatic pituitary metastasis as a presenting manifestation of silent primary malignancy were reported [7–15, 19, 25–35]. Nine of 26 cases had isolated pituitary metastasis [7–15], of which 5 had primary lung cancer [7, 9–12]. Among symptomatic patients, diabetes insipidus was the most common presenting symptom, found in 45.2% of all symptomatic pituitary metastases [1]. This resulted from the preference of metastases for the posterior pituitary [36, 37]. Rarely, central diabetes insipidus is transient due to the regeneration of neurohypophyseal neuron fibers [16]. For anterior pituitary dysfunction, 23.6% of the patients had one or more anterior pituitary hormone deficiency [1]. Corticotropin and thyrotropin deficiencies were the most common, followed by panhypopituitarism [10]. Approximately 6.3% had mild hyperprolactinemia, of which the serum prolactin levels were not higher than 149.2 ng/mL due to compression of the pituitary stalk [1].

The analysis of our patient’s hormonal profiles showed panhypopituitarism with mild hyperprolactinemia and central diabetes insipidus, corresponding to her MRI findings that both the anterior and posterior pituitary glands were involved by metastasis.

Other symptoms of pituitary metastases included visual field deficits due to compression of the optic chiasm in 27.9% of cases, ophthalmoplegia caused by cranial nerve III, IV, or VI palsy in 21.6% of cases, headache and/or post-ocular pain in 15.8% of cases, and fatigue and/or general malaise in 7.9% of cases [1].

Some clues to raise clinical suspicion of pituitary metastasis in our patient were onset of diabetes insipidus at an older age, rapidly progressive weight loss, and fatigue. These were likely symptoms of tumor burden or hypopituitarism. Also, the recent onset of bitemporal hemianopia was a clinical clue of optic chiasm compression.

The radiographic findings of pituitary metastases are usually non-specific. In MRI studies, iso- or hypointensity signals on T1W imaging and hyperintensity on T2W imaging with homogeneous enhancement after gadolinium injection are the common findings. Imaging studies may be fruitful for distinguishing metastatic disease when the pituitary stalk is thickened, and invasion of the cavernous sinus along with sclerosis of the sella turcica are seen. Nonetheless, there were no such discriminative characteristics seen in our patient except for the presence of concomitant brain lesions with the same signal intensity as that of the suprasellar mass, which offered a diagnostic clue to metastatic pituitary tumor [38].

The prognosis of patients with pituitary metastasis is poor due to the aggressiveness of the primary malignancy. The mean survival after developing a pituitary metastasis is only 6 months as in our patient, and 1-year survival is less than 10% [3].

In conclusion, metastasis to the pituitary gland is a rare and life-threatening condition that results in poor prognostic outcomes. It is difficult to diagnose, especially in patients with previously undiagnosed primary cancer. Pituitary metastasis should be considered in elderly patients presenting with new-onset diabetes insipidus with or without anterior pituitary dysfunction. MRI findings of pituitary stalk thickening, invasion of cavernous sinus, sclerotic changes around the sella turcica, and coexistence of brain lesions are also important diagnostic clues.

## Data Availability

The data that support the findings of this case report are available from the corresponding author on reasonable request.

## Abbreviations

ACTH: Adrenocorticotropic hormone
DDAVP: Desmopressin acetate
EGD: Esophagogastroduodenoscopy
FSH: Follicular stimulating hormone
FT4: Free thyroxine
IGF-1: Insulin-like growth factor 1
LH: Luteinizing hormone
MRI: Magnetic resonance imaging
SI: Signal intensity
T3: Triiodothyronine
TSH: Thyroid-stimulating hormone
TTF-1: Thyroid transcription factor-1
